# Morbidity and Mortality following Traditional Uvulectomy among Children Presenting to the Muhimbili National Hospital Emergency Department in Dar es Salaam, Tanzania

**DOI:** 10.1155/2015/108247

**Published:** 2015-06-16

**Authors:** H. R. Sawe, J. A. Mfinanga, F. H. Ringo, V. Mwafongo, T. A. Reynolds, M. S. Runyon

**Affiliations:** ^1^Muhimbili University of Health and Allied Sciences, Dar es Salaam, Tanzania; ^2^Muhimbili National Hospital, Dar es Salaam, Tanzania; ^3^University of California, San Francisco, CA, USA; ^4^Carolinas Medical Center, Charlotte, NC, USA

## Abstract

*Background*. Traditional uvulectomy is performed as a cultural ritual or purported medical remedy. We describe the associated emergency department (ED) presentations and outcomes.* Methods.* This was a subgroup analysis of a retrospective review of all pediatric visits to our ED in 2012. Trained abstracters recorded demographics, clinical presentations, and outcomes.* Results*. Complete data were available for 5540/5774 (96%) visits and 56 (1.0%, 95% CI: 0.7–1.3%) were related to recent uvulectomy, median age 1.3 years (interquartile range: 7 months–2 years) and 30 (54%) were male. Presenting complaints included cough (82%), fever (46%), and hematemesis (38%). Clinical findings included fever (54%), tachypnea (30%), and tachycardia (25%). 35 patients (63%, 95% CI: 49–75%) received intravenous antibiotics, 11 (20%, 95% CI: 10–32%) required blood transfusion, and 3 (5%, 95% CI: 1–15%) had surgical intervention. All were admitted to the hospital and 12 (21%, 95% CI: 12–34%) died. By comparison, 498 (9.1%, 95% CI: 8–10%) of the 5484 children presenting for reasons unrelated to uvulectomy died (*p* = 0.003).* Conclusion*. In our cohort, traditional uvulectomy was associated with significant morbidity and mortality. Emergency care providers should advocate for legal and public health interventions to eliminate this dangerous practice.

## 1. Introduction

Traditional uvulectomy is an excision of the uvula, usually performed by nonphysician healers. The procedure has been touted as a remedy for, or prevention of, infections associated with throat and chest and has also been completed as part of ritual practice [1–4]. Unlike the uvulopalatoplasty, which is typically performed by an otolaryngologist to treat snoring or obstructive sleep apnea [5–7], the traditional uvulectomy is performed by local healers who inherit the skills from their predecessors with no formal medical training. It is often completed without regard to recommended standards of good surgical practices [8].

In sub-Saharan Africa, traditional uvulectomy is largely driven by indigenous beliefs and cultural practices [9, 10], with many local nonphysician healers believing and preaching that the uvula is the main organ responsible for all throat and chest problems and should be removed as early as possible in childhood [11]. Despite being a common cultural practice, traditional uvulectomy has been associated with substantial number of harmful outcomes with significant morbidity and mortality largely from haemorrhage and sepsis [9, 11, 12].

In Tanzania, the practice of uvulectomy is a relatively common phenomenon [10, 13] carried out by traditional healers, most of who are not recognized by the ministry of health, despite the government stance on discouraging dangerous traditional practices. The prevalence of traditional uvulectomy in Tanzania has been estimated at 3.6% [14]. There are reports of traditional practitioners using the same instruments to perform the procedure in multiple patients without cleaning, disinfecting, or sterilizing, thus potentially exposing the children to life threatening communicable infections [15].

In this retrospective chart review study, we reviewed the records of all pediatric patients seen in the emergency department of a large national hospital during the year 2012 to describe the clinical presentations and outcomes of children presenting with complications from traditional uvulectomy.

## 2. Methods

### 2.1. Study Setting

The investigation was conducted at the Muhimbili National Hospital (MNH) Emergency Department (ED) in Dar es Salaam, Tanzania. Established in 2010, the MNH ED is the first full-capacity ED in Tanzania and is the clinical training site for the country's first emergency medicine residency program.

The department is staffed by interns (fresh graduates from medical school), registrars (registered medical practitioners each with 1–3 years of clinical experience following internship), and emergency medicine residents (all had already worked as registrars before joining the 3-year residency program). These doctors work under the clinical supervision of the locally trained emergency physician faculty with support and consultation from board-certified emergency physicians from the USA, Canada, and South Africa.

MNH is the largest tertiary care center in Tanzania and the ED serves a high acuity patient population from within Dar es Salaam as well as referral patients from throughout the country. Of the 36000 adult and pediatric patients seen each year, only 20% are discharged home from the ED. The top five categories of complaints seen in the department are trauma, infectious, mental health, neoplastic, and pregnancy-related issues [16].

### 2.2. Study Design

This was a prespecified subgroup analysis of a retrospective chart review of all children (less than 18 years old) seen in the MNH ED from 1 January 2012 to 31 December 2012. Trained physician abstractors reviewed the ED and inpatient records for all children presenting to the MNH ED during the study period. Data were recorded on a structured case report form, including basic demographics, reported initial complaints, final EMD diagnoses, and final hospital discharge diagnosis.

For this investigation, we examined all presentations associated with complications of traditional uvulectomy, including the clinical findings, treatment rendered, and patient outcomes.

### 2.3. Statistical Analysis

Data were imported into an Excel spreadsheet (Microsoft Corporation, Redmond, WA, USA), cleaned, and analyzed with StatsDirect (v 3.0.133, Cheshire, UK). We report descriptive statistics, including mean and standard deviations, medians and interquartile ranges, and counts and percentages as appropriate for the data type and distribution. Confidence intervals were calculated by the Clopper-Pearson (exact) method. Categorical values were compared between groups using the Chi Square test.

### 2.4. Ethics

The Senate research and publication committee of the Muhimbili University of Health and Allied Sciences reviewed the study protocol and granted ethical approval, including waiver of informed consent.

## 3. Results

According to the patient logs, a total of 5774 children presented to the MNH ED from 1 January 2012 to 31 December 2012. Of these, 5540 (96%) patient files were available for review and data abstraction. A total of 56 patients (1.0%, 95% CI: 0.7–1.3%) met our inclusion criteria of presentation due to complaint associated with recent traditional uvulectomy. Of those included, 30 (53.6%) of the study populations were male, with median age of 1.3 years (interquartile range: 7 months to 2 years), and 55 (98%) were below 5 years of age (Table 1).

The main presenting symptoms were cough in 46 (82%), report of fever prior to presentation in 26 (46%), and hematemesis in 21 (38%). The main physical examination findings documents in the ED charts were fever in 30 (54%), tachypnea in 17 (30%), tachycardia in 14 (25%), and hypoxia in 6 (11%) (Table 2).

The most common final ED diagnoses were pneumonia in 23 (41%), severe anemia, classified according to the World Health Organization definition as a hemoglobin measurement of less than 7 g/dL [17], in 20 (36%), and upper GI bleeding in 20 (36%). Bronchitis was the least common final ED Diagnosis (Table 3).

The most common final hospital discharge diagnoses were pneumonia in 23 patients (44%), upper GI bleeding in 24 patients (46%), Malaria in 22 patients (42%), HIV in 11 patients (21%), and severe anemia and malnutrition in 10 patients (19%). As was seen with the ED diagnoses, bronchitis was the least common hospital discharge diagnosis (Table 4).

Treatment included intravenous antibiotics for 35 patients (63%, 95% CI: 49–75%), blood transfusions for 11 (20%, 95% CI: 10–32%), and surgical interventions for 3 (5%, 95% CI: 1–15%); these surgical interventions included suture ligation for hemostasis, packing, and electrical cauterization to achieve hemostasis. Four patients (7%, 95% CI: 2–17%) died in the ED and the rest were admitted to the hospital: 42 (75%) to the general pediatric ward, 7 (13%) to the pediatric intensive care unit, and 3 (5%) to the pediatric surgical service. Eight of the admitted patients died in the hospital for a total mortality of 12 (21%, 95% CI: 12–34%) among the study cohort (Table 5). By comparison, 498 (9%, 95% CI: 8–10%) of the 5484 children with complete data who presented to the ED for reasons not related to complications of uvulectomy died (*p* = 0.003).

## 4. Discussion

Our investigation found that 1% of children presenting to the MNH ED in the year 2012 had complaints related to a recent traditional uvulectomy and nearly all were under the age of 5 years. These findings are similar to those seen in other African countries [9, 12], where the procedure is predominantly performed in the early years of life due to the belief that the elongated uvula is responsible for myriad potential maladies, including sleep disturbances, respiratory infections, and even suffocation of children in their sleep [18].

The predominant symptoms at ED presentation in our cohort were cough, difficulty in breathing, fever, and hematemesis. All the children had at least one abnormal physical finding or vital sign at the time of presentation to the emergency medicine department, with more than half of the patients presenting with fever. Other observed abnormalities included tachypnea, tachycardia, and hypoxia. We were not able to establish with certainty that the presenting symptoms of our cohort were due to complications of the procedure or from preexisting medical conditions that prompted the procedure. Regardless, the observed mortality was more than twice that of all other children presenting to the ED during the study period. Previous studies from throughout Africa have shown that cough, throat pain, dysphagia, and loss of appetite are among the most common indications for traditional uvulectomy as stated by the traditional healers [19].

The top final ED diagnoses were pneumonia, upper GI bleeding, and severe anemia. The most common hospital discharge diagnoses were similar but also included malaria. Prior work in similar settings has demonstrated that respiratory infections and malaria remain among the top causes of hospital admission [20–22]. Acknowledging the previously mentioned clinical uncertainty, it is likely that these diagnoses were responsible for the initial presentation to the traditional healers that resulted in the uvulectomy. Though respiratory infections and malaria remain among the top causes of death [23] in children under the age of 5 years, these conditions are imminently treatable and curable with timely and appropriate clinical intervention. Not only is uvulectomy ineffective but also it carries the threat of significant morbidity and mortality while delaying treatment of any significant underlying medical condition.

None of the children in this group were discharged from the emergency department, 63% received intravenous antibiotics, 20% received a blood transfusion, and 5% required immediate surgical intervention for hemorrhage control. Prior studies have reported infections and bleeding as the major complications of uvulectomy, including one in which hemorrhage was present in over 50% of the patients, with the majority requiring surgical intervention to achieve hemostasis [13, 19].

The overall mortality rate of our cohort was high (21%) with one-third of those children dying in the ED and the rest succumbing during their hospital admission. This observed morality among those presenting following a recent uvulectomy was more than double that of all other children presenting to the ED during the study period (21% versus 9%, resp., *p* = 0.003). While our study design cannot prove causation, we believe that the significantly higher mortality rate is likely due, at least in part, to a harmful traditional practice that confers no known benefit to the patient. We believe that emergency care providers should advocate for legal and public health interventions to eliminate this dangerous and unnecessary practice.

## 5. Limitations

The limitations of this study include the retrospective design and the relative paucity of clinical data available from the patients' charts. Due to the nature of the study, we were unable to reliably identify the symptoms that prompted the uvulectomy. Hence, we cannot reliably differentiate between the initial symptoms and the subsequent complications of the procedure. Likewise, we were unable to demonstrate the exact cause of death for the 12 patients who died and are unable to definitively prove whether the uvulectomy contributed to their demise.

## 6. Conclusion

In our cohort of 56 patients, representing nearly 1% of all children presenting to the MNH ED in 2012, traditional uvulectomy was associated with significant morbidity as evidenced by the need for antibiotic therapy, blood transfusions, and surgical intervention. The observed mortality rate of 21% was more than twice that of all other children presenting to the ED during the study period. These findings are alarming given that the procedure confers no clinical benefit. Emergency care providers should advocate for legal and public health interventions to eliminate this dangerous practice.

## Figures and Tables

**Table 1:** Baseline characteristics of the patients.

Variable	All (*N* = 56)^*∗*^
Age	
Overall: median (interquartile range)	1.3 years (7 months–2 years)
1–6 months	7 (13)
6 months–<1 year	18 (32)
1 year–5 years	30 (54)
>5 years	1 (2)
Gender	
Male	30 (54)
Female	26 (46)

^*∗*^Except as specified, values are counts (percentages).

**Table 2:** Clinical findings during EMD presentation.

Presenting symptoms	*N* = 56^*∗*^
Cough	46 (82)
Report of fever prior to presentation	26 (46)
Vomiting blood	21 (38)
Difficulty in breathing	12 (21)
Abdominal distension	6 (11)
Chest pain	5 (9)
Others^*∗∗*^	10 (18)

Physical findings and vital signs	*N* = 56^*∗*^

Temperature > 37.5°C	30 (54)
Tachypnea	17 (30)
Tachycardia	14 (25)
Oxygen saturation < 90%	6 (11)
Bradycardia (<80 beats/min)	5 (9)

^*∗*^Values are counts (percentages).

^*∗∗*^Others include black stool, bleeding, convulsions, and oral ulcers.

**Table 3:** Final ED diagnoses.

Diagnoses	*N* = 56^*∗*^
Pneumonia	23 (41)
Upper GI bleeding	20 (36)
Severe anemia	20 (36)
Malaria	10 (18)
HIV	8 (14)
Tuberculosis	3 (5)
Bronchitis	1 (2)

^*∗*^Values are counts (percentages). Some patients had more than one diagnosis listed.

**Table 4:** Final hospital diagnoses.

Diagnoses	*N* = 52^*∗*^
Upper GI bleeding	24 (46%)
Pneumonia	23 (44%)
Malaria	22 (42%)
HIV	11 (21%)
Severe anemia	10 (19%)
Malnutrition	10 (19%)
Septicemia	3 (6%)
Tuberculosis	3 (6%)
Bronchitis	1 (2%)

^*∗*^Values are counts (percentages). 4 patients who died in the ED were excluded. Some patients had more than one diagnosis listed.

**Table 5:** Disposition.

Disposition	All (*N* = 56)^*∗*^
General pediatric ward	42 (75)
Intensive care unit	7 (13)
Pediatric surgery	3 (5)
Died at EMD	4 (7)

Clinical outcome	All (*N* = 56)^*∗∗*^

Survived to discharge	44 (79; 66–88)
Died (both in the ward and EMD)	12 (21; 12–34)

^*∗*^Values are counts (percentages).

^*∗∗*^Values are counts (percentages; 95% confidence intervals).

## Abbreviations

ED: Emergency Department
GI: Gastrointestinal
HIV: Human immunodeficiency virus
MNH: Muhimbili National Hospital
MUHAS: Muhimbili University of Health and Allied Sciences
UK: United Kingdom
USA: United States of America.
